# Two Antarctic penguin genomes reveal insights into their evolutionary history and molecular changes related to the Antarctic environment

**DOI:** 10.1186/2047-217X-3-27

**Published:** 2014-12-12

**Authors:** Cai Li, Yong Zhang, Jianwen Li, Lesheng Kong, Haofu Hu, Hailin Pan, Luohao Xu, Yuan Deng, Qiye Li, Lijun Jin, Hao Yu, Yan Chen, Binghang Liu, Linfeng Yang, Shiping Liu, Yan Zhang, Yongshan Lang, Jinquan Xia, Weiming He, Qiong Shi, Sankar Subramanian, Craig D Millar, Stephen Meader, Chris M Rands, Matthew K Fujita, Matthew J Greenwold, Todd A Castoe, David D Pollock, Wanjun Gu, Kiwoong Nam, Hans Ellegren, Simon YW Ho, David W Burt, Chris P Ponting, Erich D Jarvis, M Thomas P Gilbert, Huanming Yang, Jian Wang, David M Lambert, Jun Wang, Guojie Zhang

**Affiliations:** China National GeneBank, BGI-Shenzhen, Shenzhen, 518083 China; Centre for GeoGenetics, Natural History Museum of Denmark, University of Copenhagen, Øster Voldgade 5-7, 1350 Copenhagen, Denmark; MRC Functional Genomics Unit, Department of Physiology, Anatomy and Genetics, University of Oxford, South Parks Road, Oxford, OX1 3QX UK; Environmental Futures Centre, Griffith University, Nathan, QLD 4111 Australia; Allan Wilson Centre for Molecular Ecology and Evolution, School of Biological Sciences, University of Auckland, Private Bag 92019, Auckland, New Zealand; Department of Biological Sciences, University of South Carolina, Columbia, SC USA; Department of Biochemistry and Molecular Genetics, School of Medicine, University of Colorado, Aurora, CO 80045 USA; Biology Department, University of Texas Arlington, Arlington, TX 76016 USA; Research Centre of Learning Sciences, Southeast University, Nanjing, 210096 China; Department of Evolutionary Biology, Uppsala University, Norbyvagen 18D, SE-752 36 Uppsala, Sweden; School of Biological Sciences, University of Sydney, Sydney, NSW 2006 Australia; Department of Genomics and Genetics, The Roslin Institute and Royal (Dick) School of Veterinary Studies, University of Edinburgh, Easter Bush Campus Midlothian, Edinburgh, EH25 9RG UK; Department of Neurobiology, Howard Hughes Medical Institute, Duke University Medical Center, Durham, NC27710 USA; Trace and Environmental DNA Laboratory, Department of Environment and Agriculture, Curtin University, Perth, WA 6102 Australia; Princess Al Jawhara Center of Excellence in the Research of Hereditary Disorders, King Abdulaziz University, Jeddah, 21589 Saudi Arabia; Department of Biology, University of Copenhagen, Ole Maaløes Vej 5, 2200 Copenhagen, Denmark; Macau University of Science and Technology, Avenida Wai long, Taipa, Macau, 999078 China; Department of Medicine, University of Hong Kong, Hong Kong, Hong Kong; Centre for Social Evolution, Department of Biology, University of Copenhagen, Universitetsparken 15, Copenhagen, DK-2100 Denmark; Department of Biology, University of Texas at Arlington, Arlington, TX 76019 USA; Bioinformatics Research Centre (BiRC), Aarhus University, C.F.Møllers Allé 8, 8000 Aarhus C, Denmark

**Keywords:** Penguins, Avian genomics, Evolution, Adaptation, Antarctica

## Abstract

**Background:**

Penguins are flightless aquatic birds widely distributed in the Southern Hemisphere. The distinctive morphological and physiological features of penguins allow them to live an aquatic life, and some of them have successfully adapted to the hostile environments in Antarctica. To study the phylogenetic and population history of penguins and the molecular basis of their adaptations to Antarctica, we sequenced the genomes of the two Antarctic dwelling penguin species, the Adélie penguin [*Pygoscelis adeliae*] and emperor penguin [*Aptenodytes forsteri*].

**Results:**

Phylogenetic dating suggests that early penguins arose ~60 million years ago, coinciding with a period of global warming. Analysis of effective population sizes reveals that the two penguin species experienced population expansions from ~1 million years ago to ~100 thousand years ago, but responded differently to the climatic cooling of the last glacial period. Comparative genomic analyses with other available avian genomes identified molecular changes in genes related to epidermal structure, phototransduction, lipid metabolism, and forelimb morphology.

**Conclusions:**

Our sequencing and initial analyses of the first two penguin genomes provide insights into the timing of penguin origin, fluctuations in effective population sizes of the two penguin species over the past 10 million years, and the potential associations between these biological patterns and global climate change. The molecular changes compared with other avian genomes reflect both shared and diverse adaptations of the two penguin species to the Antarctic environment.

**Electronic supplementary material:**

The online version of this article (doi:10.1186/2047-217X-3-27) contains supplementary material, which is available to authorized users.

## Background

Sphenisciformes (penguins), an avian order comprising six extant genera and 18 species [1], are flightless aquatic birds widely distributed in the Southern Hemisphere. Although all extant penguins have completely lost the capacity for aerial flight, they employ modified flipper-like wings in wing-propelled diving or underwater flight [2]. To be competent for an underwater life, penguins have undergone multiple morphological adaptations. For instance, penguins have developed densely packed, scale-like feathers which are good for waterproof and thermal insulation [3, 4]; their eye lens and visual sensitivity of penguins are adapted for the efficiency of underwater predation [5–7]; to overcome buoyancy force in water, penguins have developed dense bones [8] and stiff wing joints [9], and reduced the distal wing musculature [9, 10].

Penguins are the most common birds in Antarctica. Among 18 extant penguin species, eight (emperor (*Aptenodytes forsteri*), king (*A. patagonicus*), Adélie (*Pygoscelis adeliae*), chinstrap (*P. antarctica*), gentoo (*P. papua*), macaroni (*Eudyptes chrysolophus*), royal (*E. chrysolophus*) and rockhopper (*E. chrysocome*)) live in the Antarctic and sub-Antarctic areas, and two of them (Adélie and emperor) make the Antarctic continent as their major habitats [11, 12]. Antarctica is one of the most hostile environments on earth. The penguins living in Antarctica are subject to extremely low temperatures, high winds, and profound seasonal changes in the length of daylight [13]. To live in such a harsh environment, penguins have developed a complicated system in the head, wing, and legs for enhanced thermoregulation [14, 15], and an effective management of energy storage for long-term fasting [16–18]. Because of their important roles in the Antarctic ecosystem and their sensitive responses to changes in marine and Antarctic climate, penguins are also among the widely studied organisms in climate change research [19–22].

The unique morphology and remarkable life histories of penguins have attracted broad interest from scientists as well as the general public. However, most previous studies focused on ecological, physiological, behavioral, or phylogenetic aspects of their biology, whereas the molecular genetic bases of penguin adaptations remain largely unknown. As part of the avian phylogenomics project [23, 24], we sequenced the genomes of two Antarctic dwelling penguins (Adélie and emperor penguins) in order to understand the evolutionary history of penguins as well as the genomic and molecular bases of their adaptations to the Antarctic environment.

## Data description

A male emperor penguin captured from Emperor Island near Zhongshan Station and a male Adélie penguin from Inexpressible Island in the Ross Sea were used for DNA collection and sequencing. Using the Illumina Genome Analyzer platform, we generated more than 60× coverage of usable reads for each of the two penguins (Additional file 1: Table S1) [25]. The assembled draft genomes of Adélie and emperor penguins resulted in contig N50 sizes of 19.1 kb and 30.5 kb, and scaffold N50 sizes of 5.0 Mb and 5.1 Mb, respectively (Table 1). The assemblies of 1.17 Gb (54 Mb of gaps) for Adélie penguin and 1.19 Gb (72 Mb of gaps) for emperor penguin cover >85% of the estimated genome sizes of 1.25 Gb and 1.39 Gb based k-mer analysis (Additional file 2: Figure S1), respectively. The GC content of the two genomes is 41.7% and 41.8% (Table 1), towards the lower end of the range of the 48 birds sequenced (40.5-44.8%) [23].

**Table 1:** **Basic statistics of assembly and annotation of the two penguin genomes**

Species	Contig N50 length (bp)	Scaffold N50 length (bp)	Assembly size (bp)	(G + C)%	Repeats (%)	#Protein coding genes
**Adélie**	19,134	5,047,175	1,226,103,150	41.7%	6.47	15,270
**Emperor**	30,463	5,071,598	1,257,483,768	41.8%	7.38	16,070

A total of 15,270 and 16,070 protein-coding genes were annotated in Adélie and emperor penguin genomes (Table 1), respectively. We annotated 598 and 627 non-coding RNAs (ncRNA) in Adélie and emperor genomes, of which 172 (Adélie) and 180 (emperor) were microRNAs (miRNAs). 6.47% (Adélie) and 7.38% (emperor) in the two assemblies are predicted to be repetitive elements (Table 1; Additional file 3: Table S2). However, the proportions of repetitive elements in the penguin genomes should be larger, because the unassembled regions tend to contain many repetitive elements. By mapping the reads of short insert size libraries to the genome assembly of each penguin, we identified 2,559,440 and 3,410,305 heterozygous sites in emperor and Adélie, respectively. We obtained 8295 1:1 orthologs of 48 birds (including Adélie and emperor penguins) from the avian phylogenomics project [23, 24], and the CDS alignments of these orthologs were used to analyze the ratio of nonsynonymous substitution rate to synonymous substitution rate (*dN/dS*) in penguin lineages.

## Analyses

### Phylogenetic relationships of two penguins and closely related aquatic species

**Figure 1:**
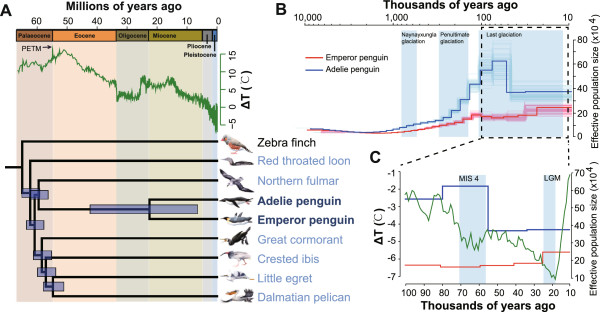
**Phylogenetic relationships and changes in effective population sizes of two penguin species. (A)** Phylogeny of two penguins and six closely related aquatic species (northern fulmar *Fulmarus glacialis*; great cormorant *Phalacrocorax carbo*; crested ibis *Nipponia nippon*; dalmatian pelican *Pelecanus crispus*; little egret *Egretta garzetta*; red-throated loon *Gavia stellata*) (blue names), and a land bird (zebra finch *Taeniopygia guttata*). The estimates of topology and divergence times are from our avian phylogenomic study [24]. Horizontal bars at each node represent 95% credibility intervals of estimated divergence times. Above the tree are the geological timescale and temperature changes over the past 65 million years, relative to the present [26]. PETM, Paleocene–Eocene Thermal Maximum. **(B)** Dynamic changes of effective population sizes (*N*_*e*_) of two penguins inferred by the pairwise sequentially Markovian coalescent (PSMC) method. The thick curves depict the estimated *N*_*e*_ values of the two penguins, and the thin curves represent PSMC bootstrapping estimates. **(C)** Enlargement of the period from 100 KYA to 10 ka in panel **(B)**. MIS 4, Marine Isotope Stage 4; LGM, last glacial maximum. Temperature change data are from [33].

In the avian phylogenomics study [24], we produced a highly resolved phylogenetic tree of 48 avian species representing almost all extant avian orders with whole-genome phylogenetic signals. The two penguins are in a relatively basal position within a clade of aquatic birds (Figure 1A). Molecular dating of 48 birds was performed in the main companion study [24] using 19 fossil calibrations, including the earlier *Waimanu* penguin fossil. We estimated that penguins diverged from their closest relatives, the order Procellariiformes (represented by the genome of northern fulmar *Fulmarus glacialis*), ~60.0 million years ago (MYA) with a 95% credibility interval (CI) of 56.8-62.7 MYA (Figure 1A). It is notable that global temperature increased dramatically during the period from 60 MYA to 50 MYA [26] (Figure 1A), and at approximately 55.0 MYA, global temperature rose by ~6°C within ~20,000 years [27]. This event, known as the Paleocene–Eocene Thermal Maximum (PETM), was associated with sea level rise and a massive benthic extinction event [28]. The global warming and massive extinction might have provided an opportunity for the offshore birds to expand their habitat to the sea, leading to the emergence of ancient penguins. The estimated divergence time between the two penguin species [24] is 23.0 MYA (95% CI: 6.9-42.8 MYA) (Figure 1A), which is slightly more ancient than that reported in a recent study using a few genes (11.7–15.4 MYA) [29].

### Analysis of effective population sizes

The population dynamics of Adélie and emperor penguins are strongly influenced by the Antarctic environment and climatic variation. Based on the heterozygous sites identified in the penguin genomes, we used the pairwise sequentially Markovian coalescent (PSMC) method [30] to infer fluctuations in the effective population sizes of the two penguins from 10 MYA to 10 thousand years ago (KYA). From 10 MYA to 1 MYA, both species had relatively small and stable effective population sizes of <100,000, and the populations expanded gradually from ~1 MYA (Figure 1B). The effective population size of the Adélie penguin appears to have increased rapidly after ~150 KYA, at a time when the penultimate glaciation period ended and the climate became warmer. This expansion is consistent with the prediction in a previous study based on mitochondrial data from two Adélie penguin lineages [31] and with the recent observations that Adélie populations expanded when more ice-free locations for nesting became available [32]. Notably, at ~60 KYA, within a relatively cold and dry period called Marine Isotope Stage 4 (MIS4) [33] in the last glacial period, the effective population size of Adélie penguins declined by ~40% (Figure 1B and C). By contrast, the effective population size of emperor penguin remained at a stable level during the same period.

It has been suggested that environmental conditions during the last glacial period were favorable to emperor penguins, because they do not require ice-free breeding grounds and are able to incubate their eggs on their feet and use an abdominal fold of skin to protect their eggs from freezing temperatures [34]. It has also been hypothesized that during the last glacial maximum (LGM, ~26.5-19 KYA, Figure 1C) [35], all penguin species except the emperor penguin were displaced from Antarctica because of the complete loss of nesting grounds and limited food sources [34]. The contrasting patterns in PSMC-based effective population sizes of the two penguins are consistent with this hypothesis. However, because the estimated divergence time between the two penguins has a large 95% CI, we cannot accurately date the population decline in the Adélie penguin. Further studies will be needed to resolve the exact timing of this decline.

### Epidermis-related genes

**Figure 2:**
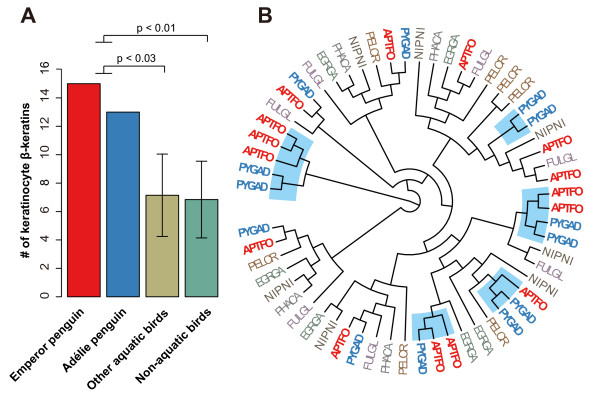
**Penguin-specific duplications of keratinocyte β**-**keratin genes. (A)** Numbers of keratinocyte β-keratin genes in the two penguins and other birds. Error bars indicate standard deviations. P-values were calculated by phylogenetic ANOVA. **(B)** Phylogenetic cladogram of keratinocyte β-keratin genes of the Adélie penguin (PYGAD, in blue), emperor penguin (APTFO, in red), and five aquatic relatives (northern fulmar, FULGL; crested ibis, NIPNI; great cormorant, PHACA; little egret, EGRGA; dalmatian pelican, PELCR). Shading in the tree indicates putative penguin-specific gene duplication events.

Penguins exhibit many unique epidermal features (including feathers) in comparison with other birds. The penguin epidermis has a thick stratum corneum, consisting of flattened heavily keratinized cells that lack basophilic nuclear remnants [36]. The feathers of penguins are short (30-40 mm), stiff, and evenly packed over the body surface to help minimize heat loss, rather than arranged in tracts as in other birds [37]. Beta(β)-keratins make up 90% of the barbs and barbules of mature feathers, and duplication and diversification of β-keratin genes are known to play important roles in the diversification of avian feathers [38, 39]. In the four subfamilies of β-keratin genes [39], we found that the numbers of keratinocyte β-keratin genes in the Adélie and emperor penguins are among the highest of all avian species (Figure 2A: emperor, 15; Adélie, 13) and only two other birds have ≥13 keratinocyte β-keratin genes (Pekin duck *Anas platyrhynchos*, 14; killdeer *Charadrius vociferus*, 13). The numbers of penguin keratinocyte β-keratin genes are significantly larger than those of other aquatic birds (7.1 ± 2.9 (mean ± S.D.); phylogenetic ANOVA, p <0.03) and non-aquatic birds (6.8 ± 2.7; phylogenetic ANOVA, p <0.01) (Figure 2A). Phylogenetic reconstruction of the keratinocyte β-keratin genes indicates the two penguin species have undergone several lineage-specific gene duplications since their divergence from their closest aquatic relatives (Figure 2B). And keratinocyte β-keratins are expressed in both skin and feathers [38, 40]. In addition, the *EVPL* gene, which encodes the protein envoplakin as a component of the cornified envelope of keratinocytes [41], has undergone positive selection in the ancestral lineage of the two penguins (branch-site model in CODEML [42], likelihood-ratio test (LRT) p = 2.32 × 10^-14^). Another gene, *DSG1*, also predicted to have evolved under positive selection in the ancestral penguin lineage (CODEML branch-site model, LRT p = 1.33 × 10^-11^), is involved in a human dermatological disorder characterized by thickening of the skin on the palms and soles [43]. The expansion of keratinocyte β-keratin genes and the positive selection on *EVPL* and *DSG1* may have contributed to generating the unique skin and feathers in penguins.

### Pseudogenes and positively selected genes involved in phototransduction

The aquatic lifestyle and marked seasonal changes in the length of daylight in Antarctica could affect the visual abilities as well as non-visual phototransduction of penguins. By comparing the genomes of 48 avian species, we found that most birds had four (tetrachromatic) classes of cone opsin genes, while Adélie and emperor penguins had only three (trichromatic) classes due to the pseudogenization of *Rh2* [23]. In addition, we found that the pinopsin gene *OPSP*, which is specifically expressed in the pineal gland and involved in circadian rhythms [44], has been pseudogenized in the two penguin species (Additional file 4: Figure S2). This provides a potential molecular explanation for a previous observation of the absence of typical photoreceptor elements in the pineal organ of gentoo penguins (*Pygoscelis papua*) [45], which is a congener of the Adélie penguin. However, the mutations leading to pseudogenization of *OPSP* in Adélie and emperor penguins were found in different codon positions (Additional file 4: Figure S2), suggesting the pseudogenization might have occurred independently in each lineage.

**Figure 3:**
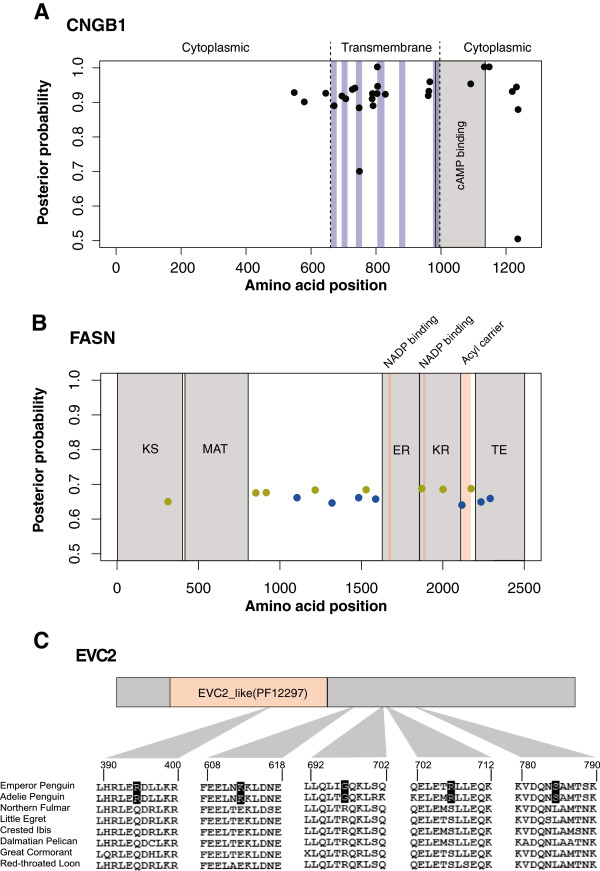
**Cases of positively selected sites and non-neutral penguin-specific amino acid changes. (A)** Positively selected sites in emperor penguin CNGB1 protein sequence. Cytoplasmic and transmembrane regions are separated by the dashed lines. Blue shading represents the membrane-spanning helix, and the cAMP binding domain is shown in grey. The posterior probabilities were calculated using BEB method in CODEML. **(B)** Positively selected sites in the FASN protein in the Adélie lineage (green dots) and the ancestral lineage (blue dots). The molecular binding domains of FASN are shown in light red, whereas the major catalytic domains are shown in grey. From left to right, beta-ketoacyl synthase (KS), acyl and malonyl transferases (MAT), enoyl reductase (ER), beta-ketoacyl reductase (KR), and thioesterase (TE). The posterior probabilities were calculated using BEB method in CODEML. **(C)** Non-neutral penguin-specific amino acid changes in the EVC2 protein. One substitution site is located in the Pfam domain EVC_like (PF12297).

Intriguingly, we detected signals of positive selection in either of the two penguin lineages on several genes involved in phototransduction (CODEML branch-site model, Additional file 5: Table S3; *CNGB1*, *MYO3A*, and *UACA* in the emperor lineage and *CRB1*, *CRY2* and *MYO3B* in the Adélie lineage), suggesting different adaptations for light transduction in the two penguins. In particular, *CNGB1*, which codes for a subunit of the cyclic nucleotide-gated cation channel in retinal rods that is important for visual perception [46], contains numerous positively selected sites in the emperor penguin lineage (Figure 3A). The different sets of positively selected phototransduction genes in the two penguins might be related to their different reproductive strategies – Adélie penguins reproduce in spring and summer with long days [47], whereas emperor penguins reproduce in the winter with short days [48].

### Positively selected genes associated with lipid metabolism

The storage of fat is critical for penguins to withstand cold and survive the long fasting periods (up to four months in male emperor penguins) [18]. We found eight, three, and four genes involved in lipid metabolism exhibiting signals of positive selection in Adélie, emperor, and their ancestral lineage of the two penguins, respectively (CODEML branch-site model, Additional file 6: Table S4). The gene *FASN*, which encodes fatty acid synthase and plays essential roles in *de novo* lipogenesis, exhibits significant positive selection in Adélie penguin (LRT p = 8.78 × 10^-5^) and the ancestral penguin lineage (LRT p = 2.77 × 10^-4^), with some of the selected sites located in its functional domains (Figure 3B, and Additional file 6: Table S4). In contrast, no evidence for positive selection on *FASN* was found in the emperor penguin lineage. As with the genes involved in phototransduction, the two penguins seem to have exploited different adaptive pathways for lipid metabolism in the course of their evolution. Because the climate prior to the divergence of the two penguins was warmer than that after their divergence, this could potentially explain why a large fraction of lipid-related positively selected genes were found in the two individual penguin lineages, rather than in the ancestral lineage.

### Forelimb-related genes with non-neutral amino acid substitutions

During their evolutionary history, the wings (or forelimbs) of penguins changed profoundly for wing-propelled diving in the water [9]. Although we did not find any *dN/dS*-based positively selected genes and pseudogenes in the ancestral penguin lineage that are linked to the forelimb adaptation, we identified 17 forelimb-related genes (of 134 genes examined) harboring non-neutral penguin-specific amino acid changes that might affect protein functions (Additional file 7: Table S5), using Protein Variation Effect Analyzer (PROVEAN) [49]. The *EVC2* gene is of particular interest because it harbors five predicted non-neutral substitutions (Figure 3C), the largest number of non-neutral substitutions among the 17 candidate genes. Mutations of *EVC2* in human can cause Ellis-van Creveld syndrome, patients of which manifest anomalies such as short-limb dwarfism, short ribs, and postaxial polydactyly [50], resembling some phenotypes in the wings of penguins. Furthermore, another gene involved in Ellis-van Creveld syndrome, *EVC*, also contains a predicted non-neutral substitution. These genes may serve as candidates for future functional studies.

## Discussion

Genome sequencing for species living in extreme environments has great potential to provide new insights into the molecular basis of adaptation to the extreme environments. For example, population genomics analysis of polar bears revealed positively selected genes associated with cardiomyopathy and vascular disease, implying important reorganization of the cardiovascular system in polar bears to adapt to the Arctic environment [51]. The genome of the Tibetan antelope exhibits signals of positive selection and gene-family expansion in genes associated with energy metabolism and oxygen transmission, suggesting high-altitude adaptation in these genes [52]. Furthermore, the recently published midge genome (*Belgica antarctica*) is the first Antarctic eukaryote genome, and has a very compact architecture which is thought to be constrained by environmental extremes in Antarctica [53]. Given their large populations and long history in Antarctica, the Antarctic penguins are an excellent model for studying how animals adapt to harsh environments, and how climate changes affect the population dynamics.

Our sequencing and initial analyses of the two Antarctic dwelling penguin species (Adélie and emperor) have shed light on the timing of penguin origin and on the effective population size changes of the two penguin species over the past 10 million years. We found evidence of associations between these biological patterns and global climate change. In particular, the contrasting patterns in effective population sizes of the two penguins during the last glacial period provide evidence for some previously proposed hypotheses about how different penguin species responded to climate change in the past. Morphological changes in the epidermis and forelimbs are critical for underwater flight in penguins, so the candidate genes that we discovered in this study are highly valuable for future functional studies. The genes involved in light transduction and lipid metabolism exhibit signals of positive selection or pseudogenization in penguins, suggesting their evolutionary responses to the extreme conditions of light and temperature in Antarctica. The pseudogenization events also show examples of relaxed constraints in the two penguins. Interestingly, we not only found shared patterns in the molecular evolution of the two penguin species, but also found distinct patterns between them, such as the genes involved in phototransduction and lipid metabolism. This implies that the diversity of molecular evolution in different penguin species deserves further investigation.

The genomic resources and the results presented here lay the foundation for further genomic and molecular studies of penguins. Given the genome sequences of two penguin species, conducting other ‘omics’ studies, such as trascriptomics and population genomics, becomes achievable in the near future. Based on the candidate genes identified in this study, future work can involve more in-depth experiments to investigate the functional roles of target genes. The divergence times of modern penguins remain somewhat unclear, and whole-genome sequencing of other penguin species will help more precise estimates to be obtained. Overall, we believe that the two penguin genomes will likely facilitate related research, such as penguin biology, avian evolution, polar biology and climate changes.

## Methods

### Genome sequencing and assembly

#### DNA preparation

The male emperor penguin was captured from Emperor Island near Zhongshan Station, and the male Adélie Penguin was collected from Inexpressible Island in the Ross Sea, Antarctica. High molecular weight genomic DNA (>100 kbp) was extracted from the peripheral venous blood of the two penguins. All the work done in this project followed guidelines and protocols for research on animals, and had the necessary permits and authorization.

#### DNA library construction and sequencing

Sequencing and assembling of the two penguin genomes both followed the whole-genome shotgun approach. All of the raw reads were generated using the Illumina Genome Analyzer platform.

1. Short insert paired-end DNA libraries (size range from approximately 200 bp to 500 bp)
  Seven DNA paired-end (PE) libraries (insert size of ~200 bp, ~350 bp and ~500 bp) (Additional file 1: Table S1) were constructed for the emperor penguin, and four DNA PE libraries (insert size of ~200 bp and ~500 bp) (Additional file 1: Table S1) were prepared for the Adélie penguin using the following steps: 1) We fragmented 5 μg of the genomic DNA by Adaptive Focused Acoustic (Covaris); 2) The DNA end was polished and a dATP was added to the 3’ end of the fragment; 3) The DNA adaptors were ligated with a dTTP overhang at the 3’ end of the fragment; 4) The ligated fragments were size-selected at 200 bp, 350 bp, 500 bp on agarose gels to yield the corresponding short insert libraries. After 10 cycles of PCR, the DNA fragments of the appropriate size were excised and purified for sequencing.
2. Long insert mate-pair DNA libraries (size range from ca. 2 kbp to 20 kbp)
  Eight mate-pair libraries were generated for the emperor penguin, and five mate-pair libraries were constructed for the Adélie penguin (Additional file 1: Table S1). Long-insert mate-pair libraries were generated based on circularization and random fragmentation. Then, 20-50 μg of genomic DNA was fragmented using the hydroshear apparatus to obtain the concentrated DNA smear. The products were end-polished and biotin-labeled with the biotinylated dNTP. After different insert-size libraries were size-selected at 2 kb, 5 kb, 10 kb, and 20 kb, 1 μg of biotinylated DNA was circularized to join the two ends, and the linear DNA was digested. The circularized DNA was fragmented randomly using the Covaris apparatus to about 400-600 bp smear, and the biotinylated DNA fragments were enriched by biotin/streptomycin on the surface of magnetic beads. End polishing, A-tailing, adaptor ligation, and PCR of 18 cycles were all performed on M-280 beads.

All DNA libraries were sequenced on Illumina GAII or Hiseq-2000 platforms in PE 50 cycles, PE 91 cycles or PE101 cycles.

#### Read filtering

For the *de novo* data we avoid mistakes from man-made or technology-system errors by a series of checking and filtering measures on reads generated by the Illumina-Pipeline.

1. Discard reads in which N or polyA sequence constitutes more than 10% of bases.
2. Discard low quality reads. Reads that have 40 bases with Q20 less than or equal to 7 for the large insert-size library data and 50 bases for the short ones were filtered.
3. Discard reads with adapter contamination. Reads with more than 10 bp aligned to the adapter sequence (allowing less than or equal to 3 bp mismatches) were removed.
4. Discard small insert-size reads in which read1 and read2 overlapped more than or equal to 10 bp allowing a 10% mismatch. Read1 and read2 are ends of one paired-end reads.
5. Discard PCR duplicates. When read1 and read2 of two paired end reads are identical, these reads were considered to be duplicates and were discarded.

In total, we obtained approximately 78.45 Gb of reads for the Adélie Penguin and approximately 80.69 Gb for the emperor penguin (Additional file 1: Table S1).

#### Estimating the genome size using k-mer frequencies

We used a method described in the panda genome paper [54] to estimate the genome sizes of the two penguins. First, we used corrected data of short insert libraries to calculate the 17-mer distribution (Additional file 2: Figure S1). We can estimate the genome size as G = K_num/K_depth, where K_num is the total number of kmers, and K_depth is the peak frequency (i.e. the mode) which occurs more often than all others (approximate sequencing depth). We otained G_Adélie_ = K_num/K_depth =38,874,560,013/31 = 1254,018,064 (bp), and G_emperor_ = K_num/K_depth =31,909,923,872/23 = 1,387,387,994 (bp).

#### Genome assembly

We used SOApdenovo2 [54] to construct contigs (kmer size =19) and scaffolds, and filled gaps in the intra-scaffolds using GapCloser (a companion program released with SOAPdenovo). Total scaffold lengths of 1,257,483,768 bp and 1,226,103,150 bp; N50 values of 5,071,598 bp and 5,047,175 bp; and, contig N50 values of 31,902 bp and 19,134 bp were obtained for the emperor and Adélie penguins, respectively (Additional file 8: Table S6). There were 80,973 and 108,883 gaps in the emperor and Adélie assemblies, covering 71,985,685 bp (5.72% of assembly size) and 54,269,351 bp (4.43% of assembly size), respectively.

Core Eukaryotic Genes Mapping Approach (CEGMA) [55] is a pipeline that can be used to evaluate the completeness of a genome assembly by annotating the 248 ultra-conserved Core Eukaryotic Genes (CEGs). The CEGMA results (Additional file 9: Table S7) revealed that the completeness of the emperor assembly is close to that of chicken and zebra finch assemblies [56, 57]. Although the Adélie assembly is not as good as the emperor, its completeness is close to the published turkey assembly [58]. One main reason for the relatively worse assembly of Adélie is that Adélie has a higher heterozygosity rate, which was also shown in the k-mer frequecy curve (Additional file 2: Figure S1). The high heterozygosity rate resulted in shorter contigs during the assembling.

We also used the whole-genome alignment between the penguin and zebra finch to assess the rearrangement events. We first generated a raw whole-genome alignment with LASTZ [59], and then used the chainNet package [60] to generate the reciprocal best net alignment. The chainNet results predicted two kinds of rearrangement events (translocation events with the “nonSyn” label and inversion event with “inv” label in netSyntenic’s output). To have a control, we also generated the reciprocal best net alignment between the previously published peregrine falcon assembly [61] and zebra finch assembly. We obtained very similar numbers of rearrangement events (relative to zebra finch) in the Adélie and emperor penguins, and the peregrine falcon (Additional file 10: Table S8).

### Genome annotation

#### Repeat annotation

We identified known transposable elements (TEs) in the two penguin genomes using RepeatMasker (version 3.2.6) [62] against the Repbase [63] library (version 15.01). We also used RepeatModeler (version 1.44) [64] to construct the *de novo* TE library for each penguin genome. RepeatMasker was used again with the *de novo* libraries to identify new TEs in both genomes. We predicted tandem repeats using Tandem Repeat Finder (TRF) [65] (version 4.00), with parameters set to “Match =2, Mismatch =7, Delta =7, PM =80, PI =10, Minscore =50, and MaxPeriod =12”. The statistics of repeat sequences of two penguins are listed in Additional file 3: Table S2.

#### Protein-coding gene annotation

The protein-coding gene annotation of the two penguins were from the avian phylogenomic project (APP hereafter) [23, 24]. Methodological details of gene annotation can be found in the avian comparative genomics study [23].

#### Non-coding RNA (ncRNA) annotation

We used tRNAscan-SE [66] to identify transfer RNA genes. The snRNA genes were predicted by INFERNAL [67] software against the Rfam database [68]. First we ran BlastN against the Rfam sequence with an E-value acceptance threshold of 1. All hits were then extended and collected as input for INFERNAL [67]. The microRNA (miRNA) genes of two penguins were predicted using two independent methods, and then combined to make a non-redundant set.

1. *Method A*
  First, hairpin sequences from miRBase [69] (V.16) were used as query sequences and then aligned to the penguin genome assemblies using WU-BLAST [70]. The outputs that matched longer than 20 bp were extended to the length of the query sequences as putative miRNA. To determine how likely the putative fragment resembled miRNA, Randfold [71] was run and sequences were retained with minimum free folding energy smaller than -15 kcal/mol and p-value smaller than 0.05. An RNAshapes [72] analysis was performed with the single-stem probability of the sequence larger than 0.9 to identify the putative miRNA, which folded into a simple stem-loop structure – more than 99% of miRNAs from miRBase had a simple stem-loop structure. Subsequently, miRNAs were retained when its seed region was 100% identical to the query sequence and the mature part was better than 90% conserved. Finally, we reserved the hit with the highest overall percent alignment identity for each locus as the miRNA sequence.
2. *Method B*
  We performed a WU-BLAST search, as in method A, to obtain putative miRNAs. To acquire precursor miRNAs, three filters were used in parallel for each hit: 1) Randfold with 1000 iterations per sequence, the outputs were filtered with Minimum Free Energy (MFE) smaller than -20 kcal/mol, and p-value smaller than 0.015; 2) PRSS [73], which works by constructing local alignments. A PRSS analysis was run with 1000 iterations in order to confirm the homology between the two sequences. The outputs were filtered with the following cutoff: E-value smaller than 10^-5^ and similarity greater than 0.65; 3) We performed a global alignment between the subject sequence (the extended hit) and query sequence (hairpin sequence from miRBase) with T-COFFEE [74], and the outputs were filtered with the similarity of >0.95. The three outputs were merged together as Set B.
3. *Combined miRNA predictions from Set A and Set B*
  The two miRNA prediction sets were combined: predicted miRNAs were considered to represent a single locus if genome coordinates overlapped on the same DNA strand, reserving the highest global identity sequence as the final precursor miRNA. Putative microRNAs were checked to remove repetitive sequence and transposable elements. Finally, 172 and 180 miRNAs were identified in Adélie penguin and emperor penguin, respectively. The statistics of annotated ncRNAs are provided in Additional file 11: Table S9.

### Phylogenetics and effective population size analysis

#### Phylogeny of two penguins and other birds

The phylogeny and divergence times for the two penguins with six closely related aquatic species (northern fulmar, great cormorant, crested ibis, dalmatian pelican, little egret, and red-throated loon) and a land bird (zebra finch) were derived from the ExaML TENT tree as described in the avian phylogenomics study [24].

#### Analysis of effective population sizes

We first identified heterozygous sites in each of the two penguin genomes. For each penguin, we used SOAPaligner [75] to map all the reads of short insert size libraries to the genome assembly, not allowing indels in the alignments. Based on the short-read alignments, we used SOAPsnp [76] to identify the heterozygous single nucleotide polymorphisms (SNPs). We performed additional screening to reduce false positives, keeping only candidates with: 1) quality score ≥20; 2) sequence depth >20; 3) the approximate copy number of flanking sequences <2; 4) at least 1 uniquely mapped read for each allele; and 5) a minimum distance between SNPs ≥5 bp.

We utilized PSMC [30] to infer the population histories of the two penguins. In order to evaluate the substitution rate of the two penguins, which is required for PSMC analysis, we aligned the two penguin genomes using LASTZ (v1.01.50) [59] with parameters “T = 2 C = 2 H = 2000 Y = 3400 L = 6000 K = 2200”. We calculated their substitution rate as: μ = (C/L)/2(T/g) =79551994/1066586108/2(22998300/5) =8.11×10^-9^ substitutions per site per generation; where C is the number of the mismatch loci between the two penguin genomes, without insertions or deletions; L is the length of the aligned sequences, without insertions or deletions; T is the divergence time between the two penguins; g = 5 is the generation time, according to [48].

After filtering SNPs located within repeat elements and putative scaffolds of the Z chromosome (based on alignment against zebra finch chromosomes), we obtained 2,559,440 and 3,410,305 SNPs in emperor and Adélie assemblies, respectively. SNPs in the assemblies of the two penguins were replaced by degenerate bases and converted to PSMC fasta-style sequence. We then ran PSMC with parameters “-N30 -t15 -r5 -p 4 + 25*2 + 4 + 6”. We also performed 100 bootstraps to estimate uncertainty in the estimates. We identified a population shrinking of ancestral Adélie penguins during the last glaciation. We used the δ18O data in [26, 33] to measure the temperature changes relative to the present; a δ18O increase of 0.22‰ is considered to be equivalent to a 1°C (1.8°F) cooling [77].

### *dN/dS*analysis

#### Ortholog identification

We obtained 8295 1:1 orthologs of 48 birds (including Adélie and emperor penguins) and the corresponding alignments from APP [23, 24], then applied the methods of ortholog assignment and alignment as described in the avian phylogenomics study [24]. CDS alignments of these orthologs were used to analyze the ratio of nonsynonymous substitution rate to synonymous substitution rate (*dN/dS*).

#### Branch model

We sought to identify genes with accelerated *dN/dS* values in the penguin lineages. We investigated three different scenarios: accelerated *dN/dS* in the Adélie lineage, in the emperor lineage, and in the ancestral lineage of the two penguins. We ran the two-ratio branch model (one *dN/dS* for the investigated branch, another *dN/dS* for other branches; set parameters “model =2, NSsites =0, fix_omega =0”) and one-ratio model (one *dN/dS* estimate for all branches, as null model; set parameters “model =0, NSsites =0, fix_omega =0”) using CODEML within the PAML package [42]. After obtaining the results of two-ratio and one-ratio models, we performed LRTs to obtain the p-values for quantifying the significance of accelerated evolution. False discovery rates (FDR) were computed using the Benjamini-Hochberg procedure to adjust for multiple testing. With a FDR cut-off of 0.05, we obtained 245, 123, and 72 genes that had accelerated *dN/dS* (fast evolving genes) in the Adélie lineage, emperor lineage and the ancestral penguin lineage, respectively. We performed Gene Ontology (GO) enrichment analysis on the three lists of genes. Only the list of fast-evolving genes in the emperor penguin exhibits enriched GO categories (Additional file 12: Table S10).

Because the results of this *dN/dS* analysis could have been affected by incomplete gene sequences or incorrect alignments, we manually checked the genes of particular interest (e.g., genes involved in vision, lipid; see related sections below). We checked the CDS alignments and removed very short gene sequences that could bias the analysis, and re-ran PAML on these revised alignments. In some cases, if the gene models appeared to be incorrectly annotated, we also performed re-annotation to obtain better gene models.

#### Branch-site model

We also ran branch-site models with CODEML in PAML to identify the genes containing positively selected sites in the penguin lineages. As with the branch models, we considered three different scenarios: the Adélie lineage, emperor lineage, and the ancestral lineage of the two penguins. The parameters for the null model were set as “model =2, NSsites =2, fix_kappa =0, fix_omega =1, omega =1”, while the parameters for the alternative model were set as “model =2, NSsites =2, fix_kappa =0, fix_omega =0”. LRT and FDR were computed as for the branch models. With an FDR cut-off of 0.05, we obtained 382, 225, and 107 positively selected genes in Adélie, emperor, and the ancestral penguin lineage, respectively. We performed GO enrichment analysis on the three lists of genes but did not find any enriched GO category.

As with the branch models, we performed an additional manual check for the genes of particular interest (see later sections). We checked whether the surrounding alignments of the positively selected sites were reliable. For suspicious alignments (which had low percent identities or many gaps), we removed problematic sequences and reran PAML on these revised alignments.

### Penguin-specific amino acid changes

We extracted the sub-alignments for two penguins and six closely related aquatic birds (*Fulmarus glacialis, Pelecanus crispus, Egretta garzetta, Nipponia nippon, Phalacrocorax carbo,* and *Gavia stellate*) from the protein alignments of 8295 orthologs [24]. Based on these alignments, we identified 14,751 penguin-specific amino acid changes (one genotype in both of the two penguins and another genotype in their close relatives) in 4922 genes, including deletions and insertions (Additional file 13: Table S11).

We used PROVEAN v1.1 [49] to predict whether a single amino acid substitution or an indel has an impact on the biological function of a protein. For each variation, PROVEAN introduced a score indicating the functional effects of this variation, and we used the default cutoff of -2.5 to determine whether the effect was non-neutral (affecting the protein function) or neutral. Under this criterion, we detected 1887 genes that harbor non-neutral amino acid changes in penguins (Additional file 13: Table S11). We also performed GO enrichment analysis on these genes. Most of the enriched GO terms were related to basic cellular functions (Additional file 14: Table S12).

### Gene family expansion and contraction

In order to investigate gene family evolution in penguins, we performed gene clustering and detected gene family expansion or contraction based on the clustering results. We chose six closely related aquatic birds (*Fulmarus glacialis, Pelecanus crispus, Egretta garzetta, Nipponia nippon, Phalacrocorax carbo,* and *Gavia stellate*) and zebra finch as the outgroup for gene clustering with the two penguins.

First, all-vs-all BLASTp for all protein sequences of nine species was performed to obtain alignments with an E-value upper threshold of 10^-5^. Then BLASTp hits were further filtered if the alignment length was smaller than 25% of the query or target length. Based on filtered BLASTp hits, “hcluster_sg” (v0.5.0) in Treefam [78] was used to cluster the genes (parameters for hcluster_sg: -w 10 -s 0.34 –m500 -b 0.1), and the resulting gene clusters were considered to be gene families. The basic statistics of gene clustering are listed in Additional file 15: Table S13.

We used CAFE [79] to identify potential gene families under significant expansion or contraction but failed to find any, probably because the difference in copy numbers in most clusters was too small. We then used the Wilcoxon rank sum test to identify gene families for which numbers in the two penguins are significantly different from those of the other seven birds. We further manually checked the significant families to ensure that the gene annotation and clustering result were correct. Finally, we obtained 10 potentially expanded and three contracted gene families (Additional file 16: Table S14). Note that the expansion of beta-keratin genes described later in this study was not predicted with this method, because the beta-keratin genes were annotated and analyzed separately.

### Analyses of genes of particular interest

#### Alpha and beta-keratins

The epidermis (including feathers) of penguins possesses many unique features compared with those of other birds. Two multigene families, alpha (α) and beta (β) keratins, play important roles in the formation of the general epidermis and epidermal appendages of birds (e.g., claws, scales, beaks, and feathers) [39, 80–83]. Therefore, we decided to investigate α and β-keratins in the two penguins and attempted to identify their differences between these two penguins and their close relatives.

The annotation of the α- and β-keratins of 48 birds were obtained from [38], as were the copy numbers of subfamilies of β-keratins in each bird. We did not find significant differences in gene numbers of α-keratins of the two penguins and the six other closely related aquatic birds (Additional file 17: Table S15). The eight investigated aquatic birds have very similar numbers of α-keratins, ranging from 32 to 36.

For the four subfamilies of β-keratins, the copy numbers of claw, scale, and feather β-keratin subfamilies tended to be affected by sequencing depth (Additional file 18: Figure S3) and the copy numbers of two penguins did not show clear differences with two high-depth sequenced close relatives (little egret and crested ibis) (Additional file 19: Table S16). However, we found significantly higher numbers of keratinocyte β-keratins in the two penguins (13 for Adélie, 15 for emperor) compared with other closely related aquatic birds (Additional file 19: Table S16). The difference in copy numbers was probably not due to sequencing depth (Additional file 18: Figure S3). The two high-depth genomes of birds that are close relatives of penguins, little egret and crested ibis, only contain six and seven keratinocyte β-keratin genes, respectively. Therefore the keratinocyte β-keratins of penguins might have undergone an independent expansion and contributed to the unique features of the epidermis of penguins. We also generated the protein sequence alignments of keratinocyte β-keratins of seven species (*Aptenodytes forsteri, Pygoscelis adeliae, Fulmarus glacialis, Pelecanus crispus, Egretta garzetta, Nipponia nippon,* and *Phalacrocorax carbo*) using Prank (v.140110) [84], and inferred the phylogenetic tree using RAxML (v8.0.14) (parameters: -m GTRGAMMA -f a -# 1000). The phylogeny inferred by RAxML is shown in Additional file 20: Figure S4.

#### Genes involved in phototransduction

In order to understand the light-sensing ability of penguins, we investigated several opsin genes in the penguin genomes, including *RH1* (rhodopsin), *RH2* (green light-sensitive), *SWS1* (violet light-sensitive), *SWS2* (blue light-sensitive), *LWS* (red light-sensitive), *OPN3* (encephalopsin), *OPN4* (melanopsin), *OPN5* (neuropsin), and *OPSP* (pinopsin). We used protein sequences for these genes from the chicken genome as our reference and used TBLASTN to find the gene locations in the genome with an E-value cutoff of 10^-5^. GeneWise was used to predict the gene structure when the alignment length was more than 50% of the query sequence. The protein sequences of predicted genes were extracted and the reciprocal best hits method was used to determine the orthology relationships with chicken opsin genes. The gene structures were then manually checked to investigate whether frameshift sites and premature stop codons were caused by errors in assembly or annotation. Among these genes, *OPSP* was found to be pseudogenized. We found two frameshifts in *OPSP* of emperor penguin, and one frameshift and one premature stop codon in *OPSP* of Adélie penguin (Additional file 21: Table S17; Additional file 4: Figure S2). We did not identify *LWS*, *SWS1,* or *SWS2* in either of the two penguin genomes. Upon searching for these three genes in other avian genomes, only a few birds were found to have these genes. The absence of these genes might be due to incomplete genome assemblies.

Based on the CODEML branch-site model test, we also identified some phototransduction-related genes that exhibit positive selection in Adélie penguin and emperor penguin lineages respectively. We further manually checked this result and excluded genes or sites with suspicious alignments. We finally found three genes with high confidence in each of the two penguins (Additional file 5: Table S3).

#### Lipid-related genes

Based on the results of CODEML branch models (FDR <0.05), we found seven, two, and four candidate lipid-related genes that had accelerated *dN/dS* in the Adélie lineage, emperor lineage, and ancestral penguin lineage. We further manually checked the results of CODEML, and redid the alignment and CODEML analysis for those with suspicious alignments. Following this process, we found six, two, and three candidate genes with high confidence (Additional file 22: Table S18).

Based on the results of CODEML branch-site models (FDR <0.05), we found eight, five, and four candidate lipid-related genes that contained positively selected sites in the Adélie, emperor, and ancestral penguin lineage. We further manually checked the results of CODEML, and redid the alignment and CODEML analysis for those with suspicious alignments. Following this process, we found eight, three, and four candidate genes with high confidence (Additional file 6: Table S4). Among these candidate genes, the most interesting is the *FASN* gene, which encodes fatty acid synthase and is essential for fatty acid synthesis. We observed positive selection signals in *FASN* in the Adélie and ancestral lineages.

#### Forelimb-related genes

The wings (or forelimbs) of penguins have been heavily modified for wing-propelled diving over the course of evolution [2]. We downloaded a list of forelimb-related genes (250 genes) from MGI (MGI id: MP:0000550 “abnormal forelimb morphology”) [85]. 59 of the 250 genes were found in 8295 orthologs mentioned in previous sections. We did not find any overlap between the 59 genes and the gene loss list and the positively selected gene list (ancestral lineage) described above. However, 11 of 59 forelimb-related genes contained non-neutral penguin-specific amino acid changes predicted to affect protein function (see the “Penguin-specific amino acid changes” section).

In addition to these 59 genes in 8295 orthologs, we generated orthologs for additional 75 genes using reciprocal best BLAST hits. Furthermore, we generated multiple sequence alignments for these 75 genes using PRANK [84]. Of the 75 orthologs, we found 17 genes harboring penguin-specific amino acid changes and six genes were predicted to harbor sites predicted to affect protein function with PROVEAN [49].

In total, we found 43 forelimb-related genes harboring penguin-specific amino acid changes in penguins, with 17 predicted to harbor non-neutral amino acid changes (Additional file 23: Table S19 and Additional file 7: Table S5).

## Availability of supporting data

The raw sequencing reads of the two penguins have been deposited in NCBI under accession numbers of SRA129317 and SRA129318. The datasets (assembly and annotation files) supporting the results of this article have been deposited in the *GigaScience* database, GigaDB [86, 87].

## Electronic supplementary material

Additional file 1: Table S1: Sequencing data generated for the Adélie and emperor penguins. (XLSX 11 KB)

Additional file 2: Figure S1: Distribution of 17-mer frequency in the sequencing reads of short-insert libraries after correction. We used all reads from the short insert-size libraries (<1000 bp). The peak depth for Adélie and emperor are 31 and 23, respectively. (PDF 15 KB)

Additional file 3: Table S2: Statistics of repeat annotation in the Adélie and emperor penguins. The predicted elements by TRF were merged with the tandem repeats predicted by RepeatMasker. “Others” refers to the repeats that can be classified by RepeatMasker, but not included by the classes above; “Unknown” refers to the repeats that can’t be classified by RepeatMasker. (XLSX 11 KB)

Additional file 4: Figure S2: The premature stop codon and frameshift sites in *OPSP*. (PDF 344 KB)

Additional file 5: Table S3: Phototransduction-related genes that exhibit positive selection in Adélie and emperor penguin lineages. “#species” indicates the number of avian species used for analysis. P-values were calculated by likelihood-ratio test based on the results of modified model A (alternative model) and corresponding null model (fixed *ω*=1). (XLSX 11 KB)

Additional file 6: Table S4: Lipid-related genes with positively selected sites in Adélie, emperor, or the ancestral lineages, predicted by PAML branch-site models. “#species” indicates the number of avian species used for analysis. P-values were calculated by likelihood-ratio test based on the results of modified model A (alternative model) and corresponding null model (fixed *ω*=1). (XLSX 12 KB)

Additional file 7: Table S5: Non-neutral penguin-specific amino acid changes in forelimb-related genes predicted by PROVEAN. (XLSX 11 KB)

Additional file 8: Table S6: Basic statistics of genome assemblies of the two penguins. (XLSX 10 KB)

Additional file 9: Table S7: CEGMA results. Prots = number of 248 ultra-conserved CEGs present in genome; %Completeness = percentage of 248 ultra-conserved CEGs present. (XLSX 10 KB)

Additional file 10: Table S8: Assessment of rearrangements based on whole-genome alignments against zebra finch assembly. (XLSX 10 KB)

Additional file 11: Table S9: Non-coding RNA genes in the genomes. (XLSX 11 KB)

Additional file 12: Table S10: Enriched GO terms in fast evolving genes in emperor penguin lineage. A cutoff of 0.05 for the FDR adjusted p-values was used. (XLSX 10 KB)

Additional file 13: Table S11: Non-neutral penguin-specific amino acid changes found in the 8295 ortholog alignments, predicted by PROVEAN. (XLSX 10 KB)

Additional file 14: Table S12: Enriched GO terms in genes harboring non-neutral penguin-specific amino acid changes. A cutoff of 0.05 for the FDR adjusted p-values was used. (XLSX 11 KB)

Additional file 15: Table S13: Basic statistics of gene clustering with Treefam. (XLSX 10 KB)

Additional file 16: Table S14: Expanded and contracted gene families in two penguins. The numbers indicate the family sizes in each species. The abbreviation of each species indicates the gene number in the family. APFTO: *Aptenodytes forsteri*, PYGAD: *Pygoscelis adeliae*, FULGL:*Fulmarus glacialis,* PELCR: *Pelecanus crispus,* EGRGA: *Egretta garzetta,* NIPNI: *Nipponia nippon,* PHACA: *Phalacrocorax carbo,* GAVST: *Gavia stellate*, TAEGU: *Taeniopygia guttata*. P-values were calculated using Wilcoxon rank sum tests. (XLSX 11 KB)

Additional file 17: Table S15: Type I and II α-keratins for two penguins and six closely related aquatic birds. (XLSX 10 KB)

Additional file 18: Figure S3: Correlation analysis between sequencing depth and copy number of each beta-keratin subfamily. The copy numbers of claw, scale, and feather β-keratin subfamilies are positively correlated with sequencing depth (p <0.05, Pearson’s test), but there is no significant correlation between sequencing depth and copy number of keratinocyte beta-keratins. (PDF 28 KB)

Additional file 19: Table S16: The numbers for four β-keratin subfamilies for the Adélie and emperor penguins and six closely related aquatic birds. (XLSX 10 KB)

Additional file 20: Figure S4: RAxML phylogeny of keratinocyte β-keratins. Adélie penguin (PYGAD, in blue), emperor penguin (APTFO, in red), and five aquatic relatives (northern fulmar, FULGL; crested ibis, NIPNI; great cormorant, PHACA; little egret, EGRGA; dalmatian pelican, PELCR). (PDF 107 KB)

Additional file 21: Table S17: Number of frameshift and premature stop codons in opsin genes. (XLSX 10 KB)

Additional file 22: Table S18: Lipid-related genes with accelerated *dN/dS* in Adélie, emperor, or the ancestral lineages, predicted by PAML branch models. “#species” indicates the number of avian species used for analysis. P-values were calculated by likelihood-ratio test based on the results of one-ratio model (null) and two-ratio model (alternative). (XLSX 11 KB)

Additional file 23: Table S19: Penguin-specific amino acid changes in forelimb-related genes. (XLSX 10 KB)

## Abbreviations

KYA: Thousand years ago
LRT: Likelihood-ratio test
MYA: Million years ago.
