# Supplementary figures and images for: Two Antarctic penguin genomes reveal insights into their evolutionary history and molecular changes related to the Antarctic environment
Source: Gigascience. 2014 Dec 12;3:27. doi: 10.1186/2047-217X-3-27 (PMC4322438; doi:10.1186/2047-217X-3-27)

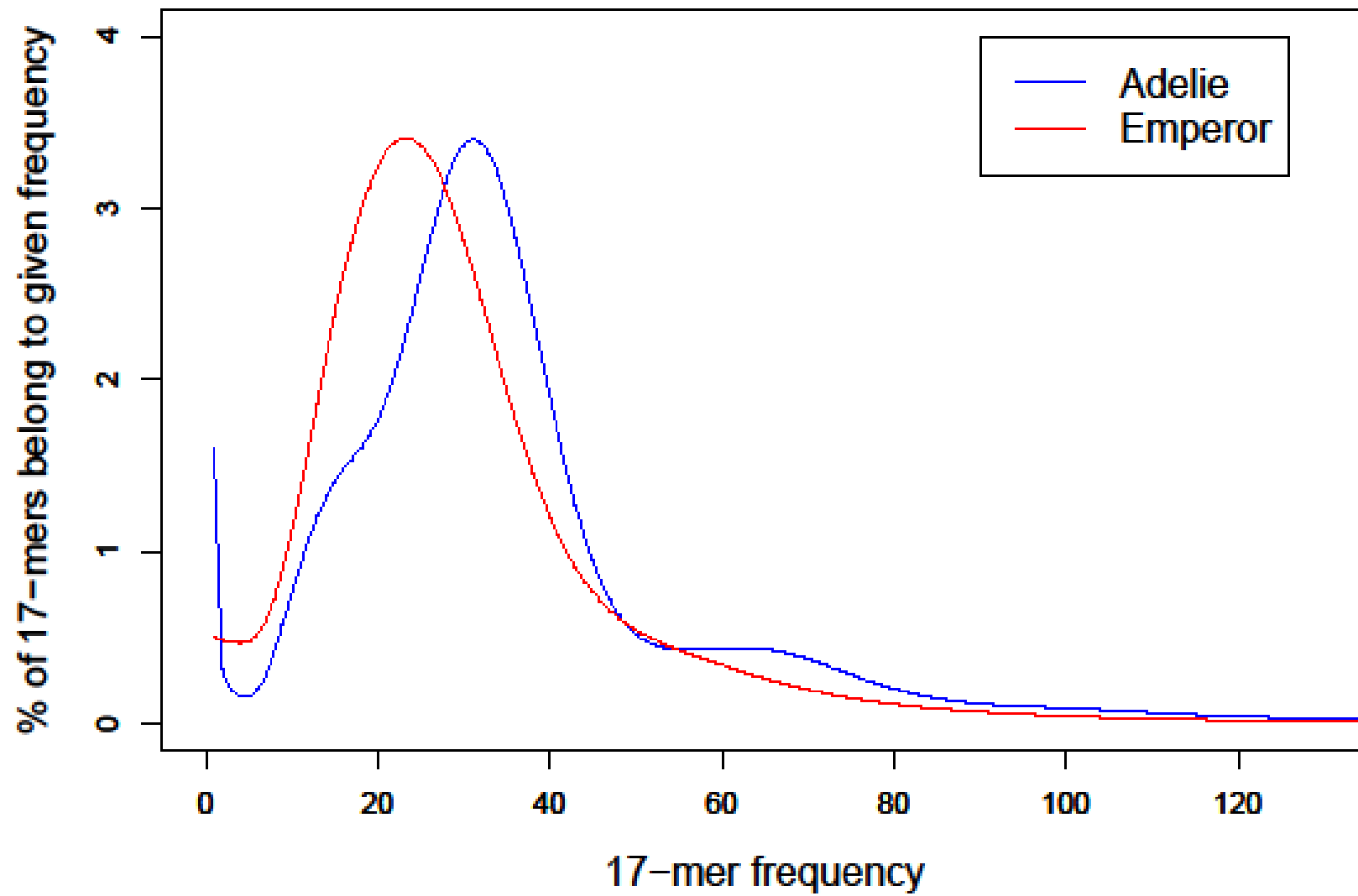

Supplement: Supplementary file 2 — Additional file 2: Figure S1: Distribution of 17-mer frequency in the sequencing reads of short-insert libraries after correction. We used all reads from the short insert-size libraries (<1000 bp). The peak depth for Adélie and emperor are 31 and 23, respectively. (PDF 15 KB) [file 13742_2014_56_MOESM2_ESM.pdf]

$r = 0.016018$  ,  $p = 0.91683$

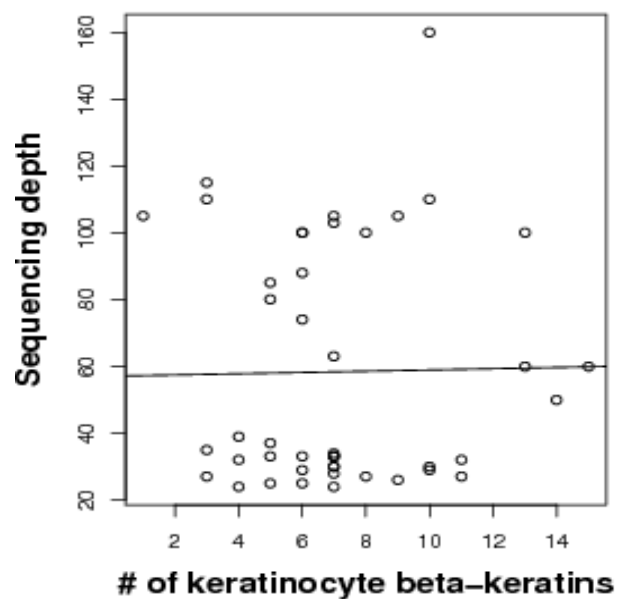

$r = 0.53176$  ,  $p = 0.00017052$

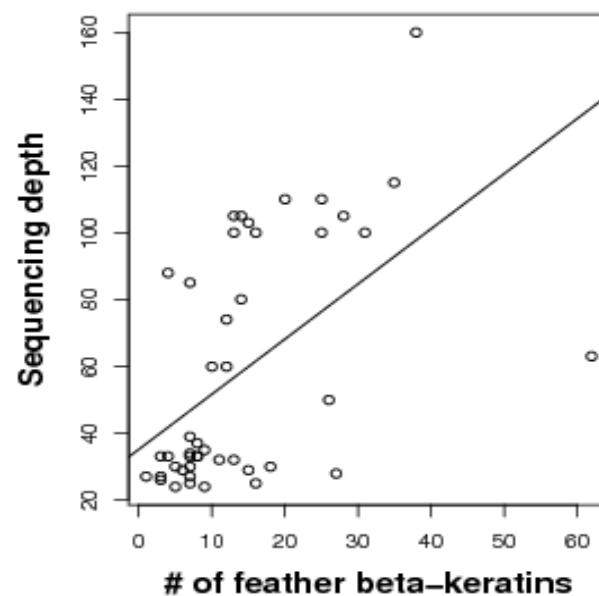

$r = 0.62538$  ,  $p = 4.3694e-06$

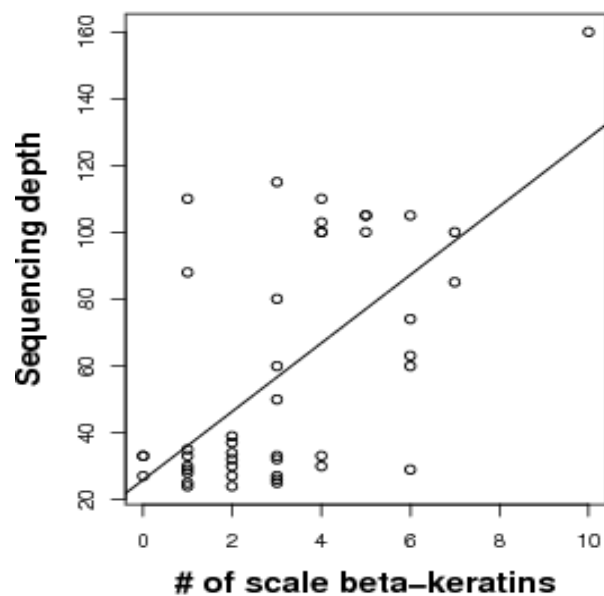

$r = 0.47482$  ,  $p = 0.00098196$

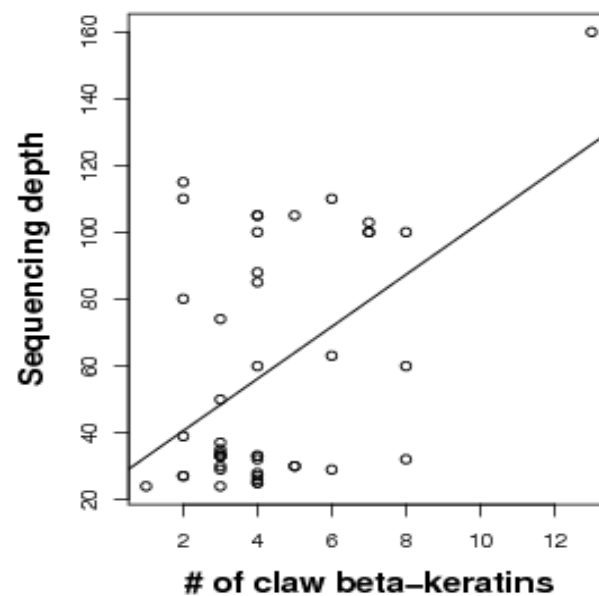

Supplement: Supplementary file 18 — Additional file 18: Figure S3: Correlation analysis between sequencing depth and copy number of each beta-keratin subfamily. The copy numbers of claw, scale, and feather β-keratin subfamilies are positively correlated with sequencing depth (p <0.05, Pearson’s test), but there is no significant correlation between sequencing depth and copy number of keratinocyte beta-keratins. (PDF 28 KB) [file 13742_2014_56_MOESM18_ESM.pdf]

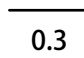

Supplement: Supplementary file 20 — Additional file 20: Figure S4: RAxML phylogeny of keratinocyte β-keratins. Adélie penguin (PYGAD, in blue), emperor penguin (APTFO, in red), and five aquatic relatives (northern fulmar, FULGL; crested ibis, NIPNI; great cormorant, PHACA; little egret, EGRGA; dalmatian pelican, PELCR). (PDF 107 KB) [file 13742_2014_56_MOESM20_ESM.pdf]
