# Supplementary material for: Two Antarctic penguin genomes reveal insights into their evolutionary history and molecular changes related to the Antarctic environment
Source: Gigascience. 2014 Dec 12;3:27. doi: 10.1186/2047-217X-3-27 (PMC4322438; doi:10.1186/2047-217X-3-27)
Supplement: Supplementary file 4 — Additional file 4: Figure S2: The premature stop codon and frameshift sites in OPSP. (PDF 344 KB) [file 13742_2014_56_MOESM4_ESM.pdf]

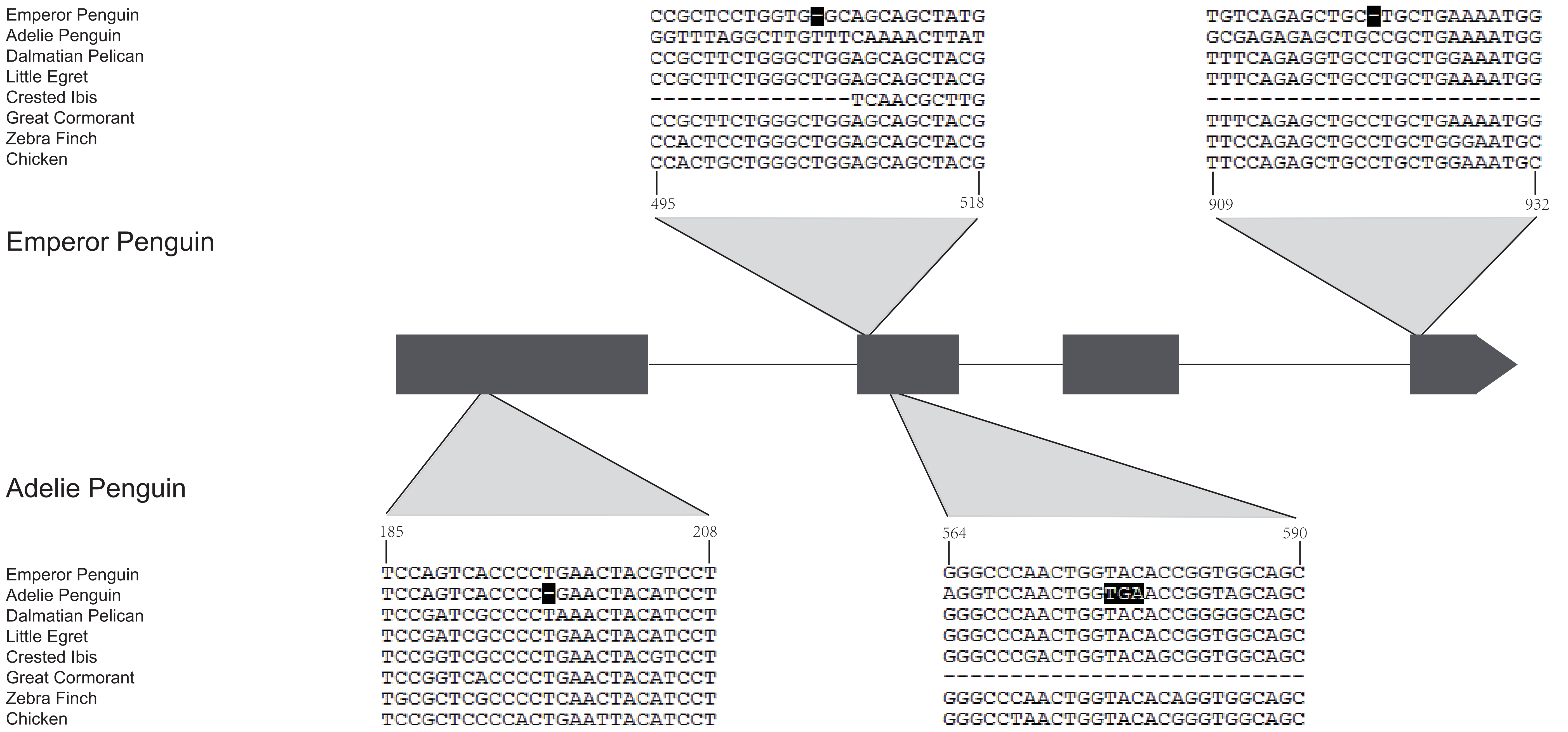
Emperor Penguin
Adelie Penguin
Dalmatian Pelican
Little Egret
Crested Ibis
Great Cormorant
Zebra Finch
Chicken
CCGCTCCTGGTG-GCAGCAGCTATG
GGTTTAGGCTTGTTTCAAAACTTAT
CCGCTTCTGGGCTGGAGCAGCTACG
CCGCTTCTGGGCTGGAGCAGCTACG
----------------TCAACGCTTG
CCGCTTCTGGGCTGGAGCAGCTACG
CCACTCCTGGGCTGGAGCAGCTACG
CCACTGCTGGGCTGGAGCAGCTACG
495
518
TGTCAGAGCTGC-TGCTGAAAATGG
GCGAGAGAGCTGCCGCTGAAAATGG
TTTCAGAGGTGCCTGCTGGAAATGG
TTTCAGAGCTGCCTGCTGAAAATGG

TTTCAGAGCTGCCTGCTGAAAATGG
TTCCAGAGCTGCCTGCTGGGAATGC
TTCCAGAGCTGCCTGCTGGAAATGC
909
932
Emperor Penguin
Adelie Penguin
185
208
564
590
Emperor Penguin
Adelie Penguin
Dalmatian Pelican
Little Egret
Crested Ibis
Great Cormorant
Zebra Finch
Chicken
TCCAGTCACCCCTGAACTACGTCCT
TCCAGTCACCCC-GAACTACATCCT
TCCGATCGCCCCTAAACTACATCCT
TCCGATCGCCCCTGAACTACATCCT
TCCGGTCGCCCCTGAACTACGTCCT
TCCGGTCACCCCTGAACTACATCCT
TGCGCTCGCCCCTCAACTACATCCT
TCCGCTCCCCACTGAATTACATCCT
GGGCCCAACTGGTACACCGGTGGCAGC
AGGTCCAACTGGTGAACCGGTAGCAGC
GGGCCCAACTGGTACACCGGGGGCAGC
GGGCCCAACTGGTACACCGGTGGCAGC
GGGCCCGACTGGTACAGCGGTGGCAGC

GGGCCCAACTGGTACACAGGTGGCAGC
GGGCCTAACTGGTACACGGGTGGCAGC
